# Effects of Implanting Exogenous Melatonin 40 Days before Lambing on Milk and Colostrum Quality

**DOI:** 10.3390/ani12101257

**Published:** 2022-05-13

**Authors:** Francisco Canto, Eloi González, José Alfonso Abecia

**Affiliations:** 1 Instituto de Investigación en Ciencias Ambientales de Aragón (IUCA), Universidad de Zaragoza, Miguel Servet, 177, 50013 Zaragoza, Spain; francisco.canto@inia.cl; 2 Departamento de Producción y Sanidad Animal y Salud Pública Veterinaria, Universidad Cardenal Herrera, Tirant lo Blanc, 7, Alfara del Patriarca, 46115 Valencia, Spain; eloigonzalezperis@gmail.com

**Keywords:** sheep, melatonin, colostrum, milk

## Abstract

**Simple Summary:**

Colostrum is the first product produced by mammals in the mammary gland immediately after parturition. It contains immunoglobulins, which are essential for the survival of the newborn. Because some evidence exists of a positive effect of melatonin on colostrum quality in sheep, we studied the effect of implanting melatonin 6 weeks before lambing in five dairy farms, and simultaneously on milk yield and quality during the first three monthly milk samplings after lambing. We compared one vs. two implants, and a control, nonimplanted group. Ewes that received a melatonin implant 40 d before lambing produced colostrum that had a higher IgG concentration than the colostrum from nonimplanted ewes, and produced more milk which, had a lower somatic cell count (SCC). The effect on SCC was prolonged if the sheep received a second melatonin implant.

**Abstract:**

The effects of exogenous melatonin implanted before lambing on the quality of colostrum and milk yield were quantified in 715 ewes. Forty days before lambing, 246 ewes (1M) received a melatonin implant; another 137 ewes (2M) received two implants, and the remaining 332 ewes (C) did not receive an implant (control). Milk analysis was based on individual monthly milk samplings (June, July, and August) after lambing. A colostrum sample was collected from 303 ewes (118 1M; 73 2M; and 112 C), and IgG concentrations were measured. Ewes implanted with melatonin had higher (*p* < 0.01) daily milk yield (DMY) in the three samplings than the C ewes. On average, 1M ewes produced more milk (*p* < 0.05) than ewes in the other two groups, and 2M ewes produced significantly (*p* < 0.05) more milk than C ewes. In the first and third controls, ewes that received two melatonin implants had a lower (*p* < 0.05) SCC than C and 1M ewes, and in the second sampling, 1M and 2M ewes had a lower (*p* < 0.01) SCC than C ewes. Ewes that received melatonin implants had a higher (*p* < 0.01) IgG concentration (21.61 ± 1.03 mg/mL) than non-implanted ewes (16.99 ± 1.13 mg/mL); 2M ewes had the highest IgG levels. In conclusion, ewes that received a melatonin implant 40 d before lambing produced colostrum that had a higher IgG concentration than the colostrum from nonimplanted ewes, and produced more milk, which had a lower SCC. The effect on SCC was prolonged if the sheep received a second melatonin implant.

## 1. Introduction

Colostrum is the first substance secreted by the mammary gland immediately after parturition, and differs from milk in its organoleptic structure, density, color, and chemical composition [1]. Colostrum is produced by colostrogenesis, in which immunoglobulin (Ig) is transferred from the maternal bloodstream to mammary secretions for a limited time within the postpartum period [2].

Factors such as age, breed, maternal nutrition, prolificacy, and health status can affect colostrum composition [3]. However, we demonstrated that treating ewes with melatonin implants in the fourth month of pregnancy increased colostrum quality as measured by IgG concentrations [4].

Melatonin is synthesized in the pineal gland, and transfers the day/night signals to the reproductive neuroendocrine axis [5], facilitated by enzymes that are controlled by the photoperiod perception. Subcutaneous implants of melatonin were used to advance the breeding season in sheep [6], and the effects of melatonin on the ovary, maternal recognition mechanism, and embryo viability were reviewed by Abecia et al. [7,8]. Carrillo-Vico et al. [9] summarized the role of melatonin in adjusting the seasonality of the immune system because it is an immunomodulatory component secreted by immunocompetent cells. In mice [10] and rats [11], melatonin has a stimulatory effect on IgG-producing cells and prevents the immunosuppression observed in aged animals because it increases IgG and IgM levels.

Melatonin implants inserted at the start of lactation do not appear to affect the milk yield of milking ewes. Melatonin implants at day 35 of lactation did not have a significant effect on milk yield or composition in Manchega and Lacaune ewes [12], and a melatonin implant at midmilking did not have a significant effect on milk yield throughout the subsequent milking period in Lacaune or Assaf dairy ewes [13]. Cosso et al. [14] did not detect a significant difference between melatonin-implanted and control Sarda ewes if the implants were inserted about two months after lambing, at the time lambs were weaned. However, the milk of ewes treated with melatonin 6 weeks before lambing presented a higher content of dry matter, protein, and fat than nontreated ewes [15].

Following experimental evidence that melatonin implants inserted once [4,15] or repeatedly [16,17] during pregnancy in ewes improved the antioxidant capacity [16] and the IgG concentration [4] in the colostrum of treated ewes, as well as the quality and the quantity [15,17] of milk, the objective of this study was to validate and quantify the effects of melatonin treatments (one or two implants) before lambing on the composition of colostrum and milk yield from ewes in a higher number of ewes, managed under normal commercial farm management procedures.

## 2. Materials and Methods

### 2.1. Animal Management and Milk Records

The experiment involved 715 Assaf ewes in their last third of pregnancy (in May) from five sheep farms in Spain. Forty days before the expected date of the onset of lambing, 246 ewes (group 1M) were implanted with a subcutaneous melatonin pellet (18 mg melatonin; Melovine, CEVA Salud Animal, Barcelona, Spain) under the left ear through a Melovine/Regulin implanter device (CEVA Salud Animal, Barcelona, Spain); another 137 ewes (group 2M) received two melatonin implants (36 mg melatonin), and the remaining 332 ewes (group C) did not receive an implant (control group). Melatonin implants provide a continuous release of melatonin and high levels of circulating plasma melatonin during the daytime for 100 days after treatment [18].

Milk analysis was based on 2145 individual monthly milk samplings (June, July, and August) after lambing. During the sampling period, data from the milk yield of each animal were collected, and a sample of milk was taken, cooled at 4 °C, then taken to the laboratory. The mean (±SEM) interval between melatonin implantation and the date of lambing, and the mean interval between melatonin implantation and the date of milk sampling are presented in Table 1. The quantity of milk produced was measured by volumetric meters inserted into the milking system and calculated using the alternated morning and afternoon records [19]. The official milk record method [19] was applied to calculate daily milk yield (DMY) through the following formula:

DMY = (Registered milk × 24)/(Time between milk records)

Milk production per month was calculated based on the ICAR method, which uses alternating monthly sampling [19]. The mean days in milk (DIM) of each experimental group on the day of milk sampling are shown in Table 1.

Immediately after lambing, a sample of colostrum was collected from 303 ewes (1M, *n* = 118; 2M, *n* = 73; and C, *n* = 112). Colostrum samples were frozen and stored at −20 °C until the analysis.

Nutritional and milking normalized management systems were applied in the farms; in particular, the sheep were raised in an intensive production system, housed permanently indoors, and, after lambing, were weaned from their lambs and milked immediately twice per day. A unifeed mixture of concentrates and forage was offered. The lambs were reared on artificial lactation until they were sold.

### 2.2. Milk and Colostrum Analyses

Fat, protein, and lactose percentage (%), and somatic cell count (SCC) were analyzed, following the IDF 020-5 [20], the FIL 105 [21], and the IDF 79-1.2/ISO 5765-1.2 [22] Standards for protein, fat and lactose content, respectively. Aliquots of each milk sample were conserved in bronopol (0.1%) to estimate the SCC by a Fossomatic 5000 (Foss Electric, Hillerød, Denmark), which we calibrated with recognized standards [23].

Colostrum concentrations of IgG were analyzed using the Calokit–Sheep Test (ZEULAB, Zaragoza, Spain) [24]. Samples were diluted to adapt the IgG concentrations to the ELISA test working range, which was read under a 450-nm absorbance Multiskan microplate reader (Labsystems, Helsinki, Finland). The minimum detection threshold for sheep colostrum was 0.82 mg/mL. The IgG concentration in colostrum samples was calculated by interpolation of a quadratic calibration curve, which we obtained by plotting the concentrations of IgG standards against the absorbance readings.

Colostrum quality was analyzed by a milk analyzer (Lactoscan SP+) that we calibrated for sheep following the manufacturer’s instructions (Milkotronic Ltd., Tsentar, Nova Zagora, Bulgaria) for measuring the fat, protein, and lactose in colostrum. Samples were 1:2 diluted before the analysis, and colostrum quality was estimated by a Brix refractometer (Deltatrak, Pleasanton, CA, USA).

### 2.3. Statistical Analysis

A multifactorial model using the least squares method of the GLM procedure in SPSS v.26 (IBM, Chicago, Il, USA) [25] was applied to compare IgG concentration in colostrum, colostrum and milk composition, and DMY, including farm and melatonin treatment as fixed effects. After that, colostrum IgG levels and colostrum and milk quality variables were statistically evaluated by an ANOVA within fixed effects. A general representation of the model is as follows: y = xb + e, where y is the N × 1 vector of records, b denotes the fixed effect in the model within the association matrix x, and e is the vector of residual effects. To assess the statistical significance of the effects of melatonin treatment (0 vs. 1 vs. 2 implants), a post hoc Fisher’s least significant difference (LSD) test was performed. A *p*-value < 0.05 was considered statistically significant.

## 3. Results

### 3.1. Milk Production and Quality

Farm (*p* < 0.0001), treatment with melatonin (*p* < 0.0001), and their interaction (*p* < 0.0001) had a significant effect on the DMY and SCC in each of the three milk samplings. Ewes implanted with melatonin had a significantly (*p* < 0.01) higher DMY in the three months than the C ewes (June: 3.29 ± 0.05 vs. 2.98 ± 0.06 kg; July: 3.37 ± 0.05 vs. 2.98 ± 0.07 kg; August: 2.89 ± 0.05 vs. 2.55 ± 0.06 kg). In addition, ewes in the 1M group produced significantly (*p* < 0.05) more milk than the ewes in the other two groups (Table 2), and 2M ewes produced significantly (*p* < 0.05) more milk than the C ewes. In the first and third samplings, ewes that received two melatonin implants had an SCC that was significantly (*p* < 0.05) lower than that of ewes in the C and 1M groups. In July, 1M and 2M ewes had significantly lower SCCs (*p* < 0.01) than the C ewes (Table 2).

**Figure 1:**
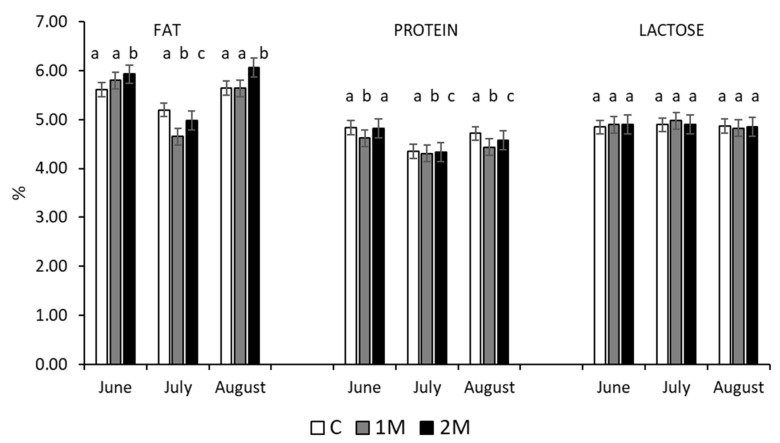
Mean (±SEM) fat, protein, and lactose content in milk samples collected in June, July, and August, of Assaf ewes that received either one (1M), two (2M), or no (C) melatonin implants before lambing (a,b,c: different superscripts indicate significant differences among groups, within the month, *p* < 0.05).

In each of the three milk sampling periods, farm (*p* < 0.001) and the interaction farm × treatment (*p* < 0.001) had a significant effect on the fat content of the milk. Farm, treatment with melatonin, and their interaction had significant (*p* < 0.001) effects on the protein content of the milk. In the first and third controls, ewes in the 2M group had a higher fat content in their milk than the ewes in the other groups (*p* < 0.05), and the groups significantly differed in the second control (Figure 1). Protein content differed among the groups in the three controls, and treatment with melatonin did not have a significant effect on the lactose content of the milk.

### 3.2. Colostrum Quality

Farm (*p* < 0.0001), treatment with melatonin (*p* < 0.0001), and their interaction (*p* < 0.0001) had significant effects on colostrum IgG concentrations. Ewes implanted with melatonin had a significantly (*p* < 0.01) higher IgG concentration (21.61 ± 1.03 mg/mL) than nonimplanted ewes (16.99 ± 1.13 mg/mL), and 2M ewes had the highest IgG levels (*p* < 0.05) (Table 3). Farm had a significant (*p* < 0.001) effect on the degrees Brix, fat, protein, and lactose content of the colostrum. The protein content of the colostrum of 2M ewes trended higher than that in the colostrum of C ewes (*p* = 0.10).

## 4. Discussion

In this experiment, melatonin implants increased milk production, reduced the SCC of the milk, and increased the IgG concentrations in colostrum, which is consistent with our earlier finding that exogenous melatonin implanted one month before lambing had a positive effect on colostrum quality as reflected in an increase in IgG concentrations [4]. In Australian Merino lambs, the melatonin treatment of ewes at midpregnancy increased survival and growth rates from birth to weaning [26]. Flinn et al. [27,28] reported an increase in the survival rate of second-born twin lambs if mothers were treated with melatonin in the second half of pregnancy, although no differences in serum IgG of the lambs were found [27]. Increased lamb survival is probably mediated by the high colostrum quality caused by exogenous melatonin and/or by an increase in brown adipose tissue and birth weight if maternal melatonin implants are inserted at day 100 of gestation [29]. Although Bouroutzika et al. [16] did not observe differences in the IgG concentration of the colostrum of pregnant ewes treated or not with melatonin, they found a better redox status in lambs born from melatonin-treated ewes, as well as higher antioxidant capacity in the colostrum of melatonin-treated ewes compared with that of the untreated ones.

Most of the evidence of the effects of exogenous melatonin on milk production in sheep has been derived from animals that were treated during lactation rather than several weeks before lambing, as in our experiment. In goats, melatonin implants inserted seven weeks before kidding had a significant effect on milk production in the subsequent lactation and improved the daily weight gain of their suckling kids [30]. Melatonin membrane receptors MT1 and MT2 are persistently expressed in the mammary glands of dairy goats throughout lactation [31], which suggests that melatonin has a direct role in the regulation of mammary physiology.

In our experiment, exogenous melatonin provided using two melatonin implants had an effect on the fat content of ewe milk in June and August, and, in a study of a meat breed of sheep, melatonin implants at lambing increased the fat content of the milk, especially at the end of lactation, and increased the growth rate of their lambs [32]. Exogenous melatonin simulates a short-day photoperiod and has significant effects on the levels of solids, protein, fat, lactose, and the fatty acids in sheep milk [33]; however, the specific role of melatonin in the regulation of milk fat synthesis, particularly in sheep, requires further study. Using bovine mammary epithelial cells, exogenous melatonin suppressed milk fat synthesis by inhibiting the signaling pathway via the melatonin-1 receptor [34]. Furthermore, exogenous melatonin significantly increased the differentiation of bovine intramuscular preadipocytes into adipocytes, in vitro, with large lipid droplets and high cellular triacylglycerol levels [35].

In our experiment and that of Cosso et al. [14], exogenous melatonin reduced the SCC in the milk of sheep. In the latter experiment, ewes had received implants about two months after lambing. Evidently, melatonin influenced the immune response in the mammary gland of the implanted ewes, although the ewes in our experiment received the exogenous hormone approximately 40 d before lambing. Yang et al. [35] reported that a subcutaneous injection of melatonin significantly reduced the SCC in the milk of cows that had subclinical mastitis, which they attributed to the anti-inflammatory and immune enhancement actions of melatonin. Furthermore, the cows that had mastitis had reduced cortisol levels and upregulated levels of IgG, IgM, lymphocytes, and neutrophils after they received melatonin administration. Blood glutathione reductase, glutathione peroxidase, and superoxide dismutase activities increased in a group of goats implanted with melatonin at the beginning of lactation [36], and SCC was significantly lower in the implanted group than in the control group at midlactation.

The reduction in somatic cells after melatonin administration is very important because the SCC of milk is a sensitive indicator of udder health (see review [37]). In addition, it is a useful characteristic for evaluating the relationship between intramammary infection and changes in milk characteristics, and the effect of a high SCC on the yield, quality, and price of milk and dairy products. Cosso et al. [14] suggested that exogenous melatonin can be a therapy for treating subclinical mastitis in sheep. In our experiment, in the 2M group, low SCC was observed until the third milk sampling (August), which was 133 days after melatonin implantation. Ewes that received one melatonin implant, however, presented an effect on SCC in the second sampling (July), 100 d after implantation. Melatonin implants can release the hormone for up to 100 d [18]; therefore, apparently, one implant can have an effect on the mammary gland up until the implant is exhausted. In ewes that received two implants, the effects of exogenous melatonin persisted for an additional month. The mechanisms involved in the reduction in SCC may be mediated either through the ability of this hormone to reduce neutrophil infiltration and the inflammatory reaction [38] or through the antioxidative effect of melatonin [39]. Moreover, it was reported that melatonin suppressed and enhanced anti-inflammatory cytokines under different pathophysiological conditions [40], which in turn may have facilitated the reduction in the SCC observed in this experiment.

## 5. Conclusions

In conclusion, ewes that received one or two melatonin implants 40 d before lambing produced colostrum that had a higher IgG concentration than that produced by nonimplanted ewes, and produced more milk, which had a lower SCC. The effect on SCC was prolonged if the sheep received two melatonin implants.

## Figures and Tables

**Table 1:** Mean (±SEM) interval between melatonin implantation and day of lambing, mean interval between melatonin implantation and day of milk sampling, and days in milk (DIM) for the milk sampling of Assaf ewes that received either one (1M), two (2M) or no (C) melatonin implants 40 days before lambing.

Group	Number of Ewes/Farm	Interval Implant–Lambing (Days)	Interval Implant–Milk Sampling (Days)			DIM (Days)		
	F1 F2 F3 F4 F5		June	July	August	June	July	August
C	35 87 27 30 153	--	--	--	--	12.6 ± 0.4	54.7 ± 1.1	95.7 ± 1.0
1M	28 91 33 23 41	44.6 ± 0.6	57.3 ± 0.8	100.3 ± 1.0	134.0 ± 0.8	15.9 ± 0.5	55.8 ± 0.9	89.5 ± 0.9
2M	32 40 34 20 41	40.0 ± 0.7	48.1 ± 0.9	96.9 ± 1.7	133.0 ± 1.4	12.0 ± 0.7	57.4 ± 1.4	93.5 ± 1.3

**Table 2:** Mean (±SEM) daily milk yield (DMY) and somatic cell count (SCC) of Assaf ewes that received either one (1M), two (2M), or no (C) melatonin implants 40 days before lambing (a,b,c: different superscripts indicate significant differences among groups, within the month, *p* < 0.05).

Group	DMY (kg)			SCC (×1000)		
	June	July	August	June	July	August
C	2.98 ± 0.06 ^a^	2.99 ± 0.07 ^a^	2.55 ± 0.06 ^a^	1597 ± 213 ^a,b^	1420 ± 216 ^a^	1355 ± 173 ^a,b^
1M	3.36 ± 0.07 ^b^	3.47 ± 0.07 ^b^	2.98 ± 0.06 ^b^	1491 ± 230 ^a^	884 ± 133 ^b^	1220 ± 183 ^a^
2M	3.19 ± 0.08 ^c^	3.21 ± 0.07 ^c^	2.75 ± 0.08 ^c^	960 ± 202 ^c^	623 ± 159 ^b^	715 ± 187 ^c^

**Table 3:** Mean (±SEM) concentration of IgG, degrees Brix, and fat, protein, and lactose content in colostrum immediately collected after lambing from Assaf ewes that received either one (1M), two (2M), or no (C) melatonin implants before lambing (a,b,c: different superscripts indicate significant differences among groups, *p* < 0.05) (x,y: different superscripts indicate differences among groups, *p* = 0.10).

Group	IgG (mg/mL)	°Brix (%)	Fat (%)	Protein (%)	Lactose (%)
C	16.99 ± 1.13 ^a^	21.70 ± 0.49 ^a^	7.02 ± 0.34 ^a^	7.06 ± 0.11 ^a,x^	6.71 ± 0.11 ^a^
1M	19.88 ± 1.39 ^b^	22.06 ± 0.38 ^a^	7.09 ± 0.28 ^a^	7.18 ± 0.13 ^a^	6.89 ± 0.13 ^a^
2M	24.41 ± 1.46 ^c^	22.57 ± 0.42 ^a^	7.65 ± 0.29 ^a^	7.39 ± 0.16 ^a,y^	7.06 ± 0.16 ^a^

## Data Availability

The data presented in this study are available on request from the corresponding author.
